# The role of ferro-aging genes in periodontitis: novel insights based on bioinformatics analysis

**DOI:** 10.3389/fcimb.2026.1875267

**Published:** 2026-10-08

**Authors:** Poyi Chu, Danhua Wu, Jianwen Deng, XuFeng Chen

**Affiliations:** 1 Medical Center of Stomatology, The First Affiliated Hospital, Jinan University, Guangzhou, China; 2 Zeren Dental Clinic (Guangzhou) Co., Ltd., Guangzhou, China

**Keywords:** celldeath, imune cell, LASSO, periodontitis, WGCNA

## Abstract

**Background:**

The concentration of iron ions in gingival crevicular fluid and local periodontal tissues is significantly elevated in periodontitis (PD), presenting a disorder of iron metabolism characterized primarily by iron overload. Excessive free iron can continuously catalyze the generation of reactive oxygen species through the Fenton reaction, providing sustained chemical driving force for lipid peroxidation, thereby constituting a key biochemical basis for the initiation of ferro-aging. However, the specific role of ferro-aging in the pathogenesis of PD and its molecular regulatory network remains unclear.

**Methods:**

The study obtained PD-related transcriptomic datasets from the Gene Expression Omnibus database. By intersecting the ferro-aging-related gene set (FAG) with differentially expressed genes (DEGs), ferro-aging-related differentially expressed genes (FAGDEGs) in PD were screened. Subsequently, a comprehensive application of Weighted Gene Co-expression Network Analysis (WGCNA) and two machine learning algorithms—Random Forest (RF) and Least Absolute Shrinkage and Selection Operator (LASSO) regression—was employed to further screen key ferroptosis-associated genes (KFAGs). Receiver Operating Characteristic (ROC) curve was used to evaluate the diagnostic performance of KFAGs, and experimental validation was performed using quantitative real-time polymerase chain reaction (RT-qPCR). Additionally, immune cell infiltration analysis was conducted to explore potential associations between the expression levels of KFAGs and immune cell types.

**Results:**

This study identified a total of 1379 DEGs, and after intersecting them with FAG, 11 FAGDEGs were obtained. Following screening by WGCNA and machine learning algorithms, 4 KFAGs were ultimately identified: IRF1, SLC1A5, PDE4B, and IL1B. ROC curve analysis results showed that the Area Under the Curve (AUC) of the above four genes was greater than 0.6, indicating their good potential value in the diagnosis of PD. RT-qPCR experimental results demonstrated that the expression trends of these four KFAGs in PD were consistent with the database analysis results. Immune cell infiltration analysis further revealed significant correlations between the expression levels of KFAGs and the abundance or activation status of various immune cell subsets.

**Conclusion:**

The results of this study indicate that IRF1, SLC1A5, PDE4B, and IL1B are key biomarkers associated with the ferro-aging process in PD. This finding provides new molecular evidence and research directions for further elucidating the synergistic role of iron metabolism disorders and ferro-aging in the pathogenesis of PD.

## 1 Introduction

Periodontitis (PD) is a major cause of tooth loss in adults, characterized by progressive destruction of the periodontal supporting tissues (including the gingiva, periodontal ligament, cementum, and alveolar bone) (Könönen et al., 2019). In recent years, the role of cellular senescence in the pathogenesis of PD has gained increasing attention, with senescent cells accumulating in periodontal tissues and exacerbating local inflammation and tissue destruction through the secretion of the Senescence-Associated Secretory Phenotype (SASP). The pathological progression of PD is not driven by a single mechanism; the complex interplay between iron homeostasis dysregulation and cellular senescence constitutes a key pathological axis. Clinical and animal studies have observed a tendency for iron metabolism disorders in PD (Wu et al., 2019; Lösser et al., 2024; Torres et al., 2024). This iron homeostasis imbalance not only affects the whole body but also exacerbates tissue destruction in the local periodontal microenvironment through multiple mechanisms. In the PD microenvironment, iron overload directly impairs the function of immune cells, accelerates their cellular senescence, thereby weakening host defense capabilities and exacerbating chronic inflammation. For example, ferroptosis—a novel form of cell death driven by iron-dependent lipid peroxidation—has been found to be aberrantly activated in various senescence-related diseases. In the PD microenvironment, persistent inflammation and oxidative stress may simultaneously induce senescence and ferroptosis in gingival fibroblasts and periodontal ligament cells (Masi et al., 2011; Xia et al., 2017; Zhao et al., 2020), forming a vicious cycle that jointly drives disease progression. Therefore, in-depth analysis of the interaction network of the “iron-senescence-periodontitis” pathological axis and the identification of biomarkers with high specificity and sensitivity from it are of great significance for early disease warning, precise intervention, and efficacy evaluation.

Ferro-aging is an emerging cross-disciplinary biological concept that integrates the core features of two cell fates: Ferroptosis and Senescence. Although ferroptosis and ferro-aging share the iron-dependent and lipid peroxidation pathways, they exhibit essential differences in time scale, biological outcomes, and intervention strategies. Ferro-aging is a chronic, progressive aging program triggered by iron overload, driven by Acyl-CoA Synthetase Long-Chain Family Member 4 (ACSL4) protein, and mediated by lipid peroxidation, characterized by sublethal, persistent iron accumulation and oxidative stress, spanning the entire senescence process of primates and occurring continuously across tissues (Liu et al., 2026). In contrast, ferroptosis is an acute, regulated form of cell death characterized by iron-dependent accumulation of lipid peroxides. In recent years, ferroptosis has been confirmed to be widely involved in the pathological processes of inflammatory diseases, including PD, but it may not fully explain the chronic senescence characteristics observed in chronic inflammatory diseases. Studies have found significant accumulation of senescent cells in PD lesional tissues (Kim et al., 2025). Additionally, abnormal iron accumulation and elevated expression of lipid peroxidation products have been observed in the periodontal tissues, serum, and gingival crevicular fluid of PD patients (Veljovic et al., 2022; Zhang et al., 2025). Since ferroptosis and ferro-aging share upstream signals, there is a theoretical basis for their interaction. In the context of PD, while iron homeostasis disruption triggers ferroptosis, it may also create conditions for the occurrence of ferro-aging.

Existing research has almost entirely focused on the acute lethal events of ferroptosis, lacking specific molecular indicators that can distinguish ferro-aging from ferroptosis. This prevents us from effectively identifying patient subtypes driven by ferro-aging in PD from public transcriptome data, nor can we quantify the association between ferro-aging-related genes and PD progression or systemic aging. Based on this, this study aims to screen and validate characteristic biomarkers of ferro-aging in PD through bioinformatics mining combined with experimental validation, in order to distinguish ferro-aging from classical ferroptosis events and provide a new biomarker system for revealing the independent driving mechanisms of chronic functional decline in PD patients.

## 2 Materials and methods

### 2.1 Data sources and processing

Download the human PD-related datasets GSE16134 and GSE23586 from the Gene Expression Omnibus (GEO) (https://www.ncbi.nlm.nih.gov/geo/). Both datasets are microarray matrix data, with the chip platform being GPL570, and the tissue source being gingival tissue. In GSE16134, periodontitis was defined as PD ≥ 4 mm, AL ≥ 3 mm, and BoP positive, while healthy controls were defined as PD ≤ 4 mm, AL ≤ 2 mm, and BoP negative. This dataset comprised 241 PD samples and 69 healthy controls from systemically healthy, non-smoking individuals without diabetes or other systemic diseases. Participants ranged in age from 13 to 76 years (mean 39.9 years), with 50.8% male. GSE23586 included 3 PD samples and 3 healthy control samples using comparable criteria. Both datasets were used as the test set. GSE10334 contains 183 PD samples and 64 healthy control samples, and it was selected as the validation set.

Standardization and normalization were performed on all datasets using the “limma” and “sva” packages in R software. GSE16134 and GSE23586 datasets were merged, and the ComBat function was used to eliminate batch effects in the data. The results of batch effect removal were visualized through principal component analysis (PCA) plots.

Ferro-aging-related gene set (FAG) data were obtained from previous studies, comprising a total of 95 genes (**Supplementary Material 1**).

### 2.2 Identification of PD differential gene expression

In the combined dataset of GSE16134 and GSE23586, the differentially expressed genes (DEGs) analysis was performed between the PD group and the control group using the “limma” package in R software. The screening criteria were defined as: |log2 FC| > 0.5 and P value < 0.05. The “ggplots” and “pheatmap” packages were used to visualize the DEGs results by generating volcano plots and heatmaps, respectively.

### 2.3 Identification and functional enrichment analysis of differentially expressed genes related to ferro-aging

After intersecting the obtained DEGs with FAG, the ferro-aging-related differentially expressed genes (FAGDEGs) can be identified. Furthermore, to understand the functions of FAGDEGs and the enrichment of their functional pathways, we performed Kyoto Encyclopedia of Genes and Genomes (KEGG) and Gene Ontology (GO) analyses on the obtained FAGDEGs. The GO enrichment analysis mainly includes the enrichment of genes in three categories: biological process (BP), cellular component (CC), and molecular function (MF). The screening criteria based on enrichment analysis were set as (p<0.05 and q-value<0.25), and the results were visualized using the “clusterProfiler” package, “ggplot2” package, and “enrichment” package in R software.

### 2.4 WGCNA network analysis and identification of key genes

To identify gene modules associated with PD, this study used the “WGCNA” package in R software for WGCNA analysis.

The top 50% of genes with the highest variance were selected for analysis. Samples were preprocessed by removing genes with missing values and abnormal values. The soft threshold β was set in the range of 1 to 15, and the minimum β satisfying the scale-free topology fitting index R² ≥ 0.9 was selected. Based on the selected β, an adjacency matrix was constructed and converted into a topological overlap matrix (TOM). After identifying preliminary modules through hierarchical clustering and dynamic tree cutting, similar modules were merged using a threshold of 0.25 for the dissimilarity of module eigengenes, and modules with fewer than 50 genes were merged into the most similar module. Module genes with a correlation to PD ≥ 0.3 and p < 0.05 were selected, and Venn diagrams were used to identify the intersection genes between FAGDEGs and WGCNA module genes, which were subsequently used for further analysis.

### 2.5 Machine learning to determine the final core key genes

Least absolute shrinkage and selection operator (LASSO) and random forest (RF) algorithms were used to identify the optimal diagnostic genes for PD.

First, based on the R language glmnet package, the optimal penalty parameter λ was determined through ten-fold cross-validation, and features with non-zero coefficients were screened using L1 regularized LASSO regression (α = 1, family = “binomial”). Second, the randomForest package was used to construct a random forest model, with the number of decision trees set to 1000, and the importance of each feature was evaluated by mean decrease accuracy (or Gini coefficient), retaining features with importance scores above the median. Finally, the intersection genes of the features screened by the two methods were taken as the final key variables for subsequent modeling and analysis.

### 2.6 Construction of a diagnostic nomogram model and validation of core key genes

After integrating key genes jointly screened by LASSO and RF, the lrm function in the rms package was used to fit a logistic regression model, with PD as the outcome variable. Based on this logistic regression model, a nomogram was constructed to visually predict the risk of non-adherence. The C-index (concordance index) was used to evaluate the model’s discriminative ability, calculated via the rcorrcens function. ROC curves were plotted using the pROC package, and the area under the curve (AUC) was calculated to further quantify discrimination. The model’s calibration was assessed using calibration curves, with predicted probabilities compared to actual observed proportions through 1000 bootstrap resamples (calibrate function). Finally, decision curve analysis (DCA) was performed using the decision_curve function to evaluate the clinical net benefit of the nomogram across different threshold probability ranges.

To further validate the expression levels of core genes, independent sample t-tests were used in the test sets (GSE16134 and GSE23586) to compare expression differences between the control group and the PD group, and box plots with jitter points were drawn for visualization. Significance levels were denoted as * p < 0.05, ** p < 0.01, *** p < 0.001. Subsequently, ROC curve analysis was performed using the pROC package to calculate the area under the curve (AUC) for each gene, and ROC curves were plotted in different colors to assess their discriminative ability. The final obtained genes were identified as key ferro-aging-related genes (KFAGs) associated with PD.

### 2.7 Immune cell infiltration analysis

The single-sample gene set enrichment analysis (ssGSEA) was used to quantify the level of immune cell infiltration. The Wilcoxon rank-sum test was employed to compare the differences in the infiltration scores of 22 immune cell types between the control group and the PD group, and box plots were generated using the ggplot2 and ggpubr packages for visualization. To explore the association between core genes and immune cell infiltration, Spearman rank correlation analysis was performed using the psych package, and multiple comparisons were corrected by the false discovery rate (FDR). The results of the correlation analysis were presented in a dot plot format, where the size and color of the dots represent the correlation coefficients, and significance levels were annotated (* p < 0.05, ** p < 0.01, *** p < 0.001).

### 2.8 Clinical sample collection

This study has been approved by the Ethics Committee of the First Affiliated Hospital of Jinan University (Approval KY-2025-187). Gingival tissue samples were obtained from a total of 6 subjects (3 females and 3 males, aged 25 to 55 years). All participants were systemically healthy, with no concomitant diseases (including Diabetes Mellitus) affecting periodontal status. They were all Non-smokers and had not received any systemic antibiotic or anti-inflammatory treatment for at least 6 months prior to sampling. Gingival tissues from the control group (n=3) were collected from intraoperative resected samples of periodontally healthy individuals undergoing periodontal crown lengthening surgery. Gingival tissues from the periodontitis group (n=3) were collected from the internal bevel incision area of teeth requiring periodontal flap surgery, or from gingival tissues of teeth with no retention value. All samples originated from free gingiva, including the epithelial layer and subepithelial connective tissue. The diagnosis and inclusion criteria for the control and PD groups were based on previous literature (Lin and Jin, 2021), with periodontitis defined as probing depth (PD) ≥ 4 mm, attachment loss (AL) ≥ 3 mm, and bleeding on probing (BoP) positive, and healthy controls defined as PD ≤ 4 mm, AL ≤ 2 mm, and BoP negative. All participants met these diagnostic criteria and provided written informed consent prior to enrollment.

### 2.9 RT-qPCR

Total RNA was extracted using RNAiso Plus (Takara Bio, Inc.) and reverse transcribed to generate cDNA using the Hifair^®^ II 1st Strand cDNA Synthesis Kit (Yeasen, Shanghai, China; Cat. no. 11119ES60). Quantitative Reverse Transcription Polymerase Chain Reaction (RT-qPCR) was performed according to the manufacturer’s instructions using the ChamQ Blue Universal SYBR qPCR Master Mix (Vazyme#Q312). The relative quantification results of the target genes in each sample were calculated using the 2^-ΔΔCt method. The RT-qPCR primers are listed in **Supplementary Material 1**.

### 2.10 Statistical analysis

Statistical analysis was performed using R software (version 4.4.2). For comparisons between two groups, the T-test was used for data that followed a normal distribution and had homogeneity of variance; Welch’s t’ test was used for data that followed a normal distribution but had heterogeneity of variance; and the Wilcoxon rank-sum test was used for data that did not follow a normal distribution. Linear correlations between variables were analyzed using the Pearson correlation coefficient. The significance level was set at p < 0.05.

## 3 Result

### 3.1 Dataset preprocessing and merging

By using the “sva” package in R software, the two PD datasets, GSE16134 and GSE23586, were integrated and subjected to standardization and batch effect removal, resulting in a merged GEO dataset. The PCA results (**Figure 1**) indicate that after the batch effect removal procedure, the sample batch effect of the merged PD dataset was significantly reduced.

**Figure 1:**
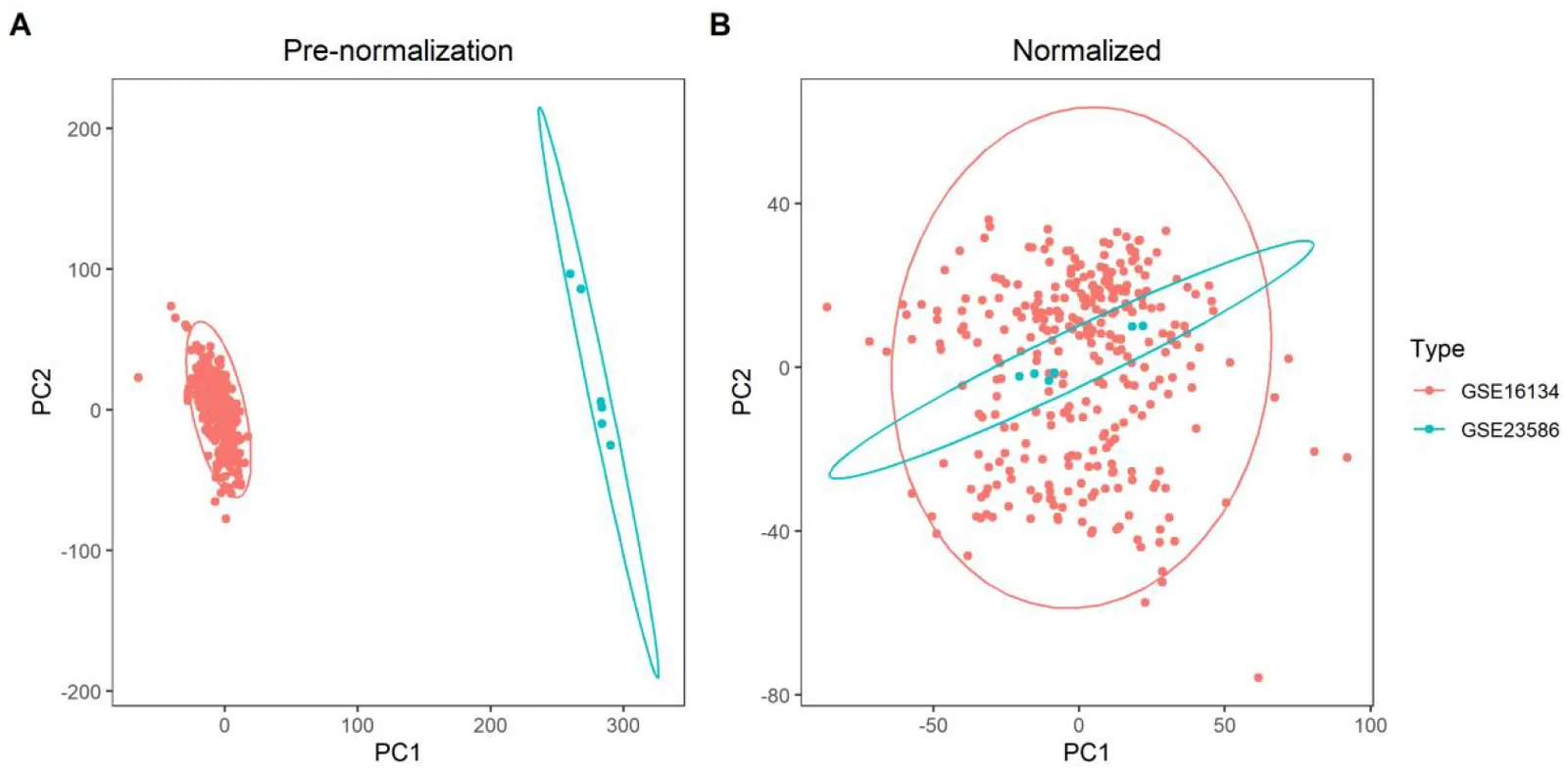
Remove batch effects from the dataset. **(A)** PCA plot before batch effect removal. **(B)** PCA plot after batch effect removal.

### 3.2 Differential gene expression analysis

By using the “limma” R package to identify differential expression in the merged PD dataset, a total of 1379 DEGs were identified. Among them, 815 genes were upregulated, and 564 genes were downregulated in PD. The relative expression levels and distribution of these genes in all samples were visualized through volcano plots (**Figure 2A**) and heatmaps (**Figure 2B**), where the depth of color indicates the level of gene expression. Typically, red represents high expression and blue represents low expression, with darker colors indicating higher expression levels.

**Figure 2:**
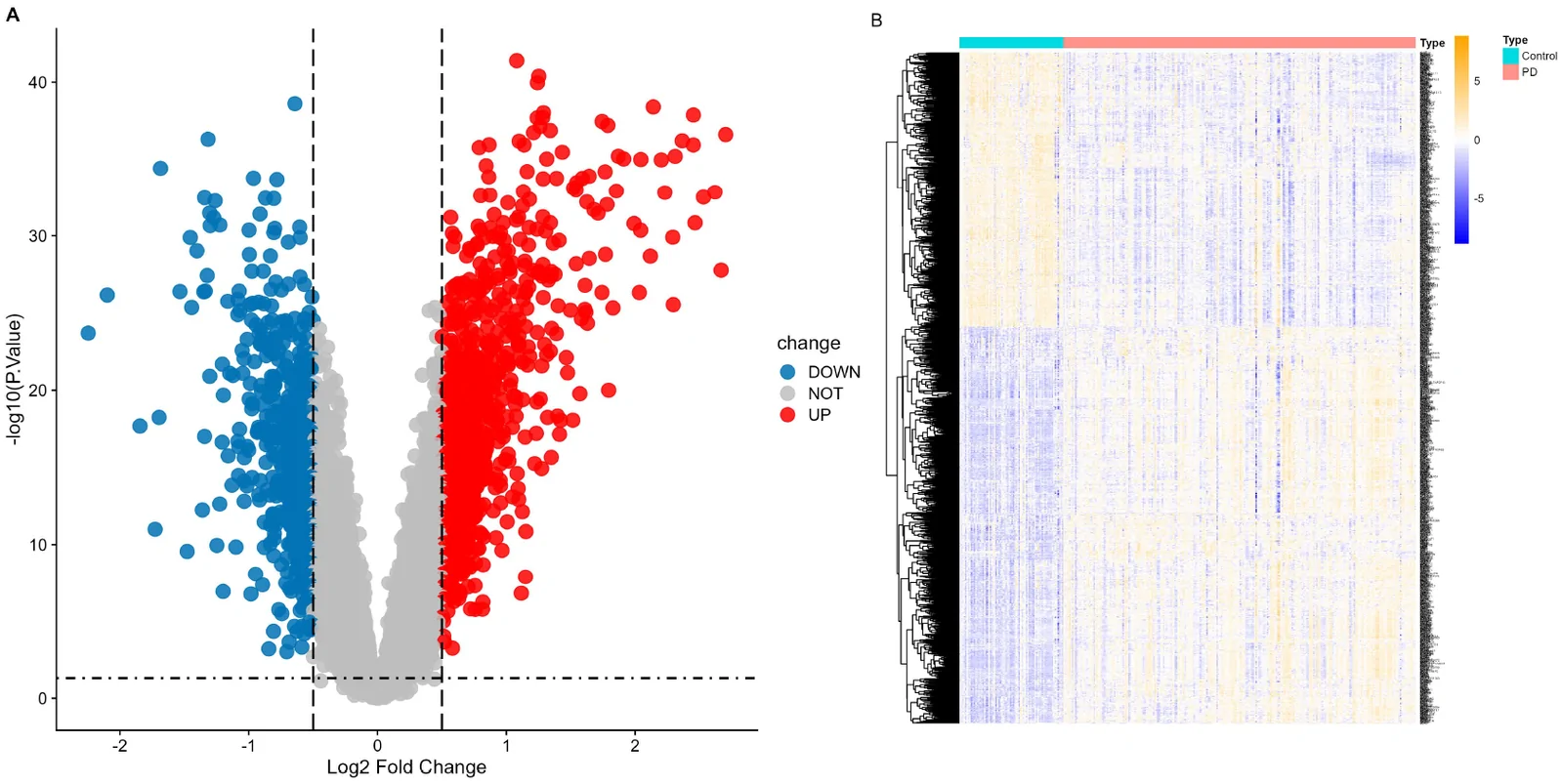
Identify differentially expressed genes. **(A)** Volcano plot of differential gene expression. **(B)** Correlation heatmap of differential gene expression. PD, periodontitis.

### 3.3 Identification and functional enrichment analysis of FAGDEGs

Intersection analysis of 95 FAG and 1379 DEGs (**Figure 3**) identified a total of 11 FAGDEGs, namely RUNX3, IL1B, SLC1A5, IGFBP7, IRF1, PDE4B, CTSB, TXNIP, EGR1, IL6, and PTGS2.

**Figure 3:**
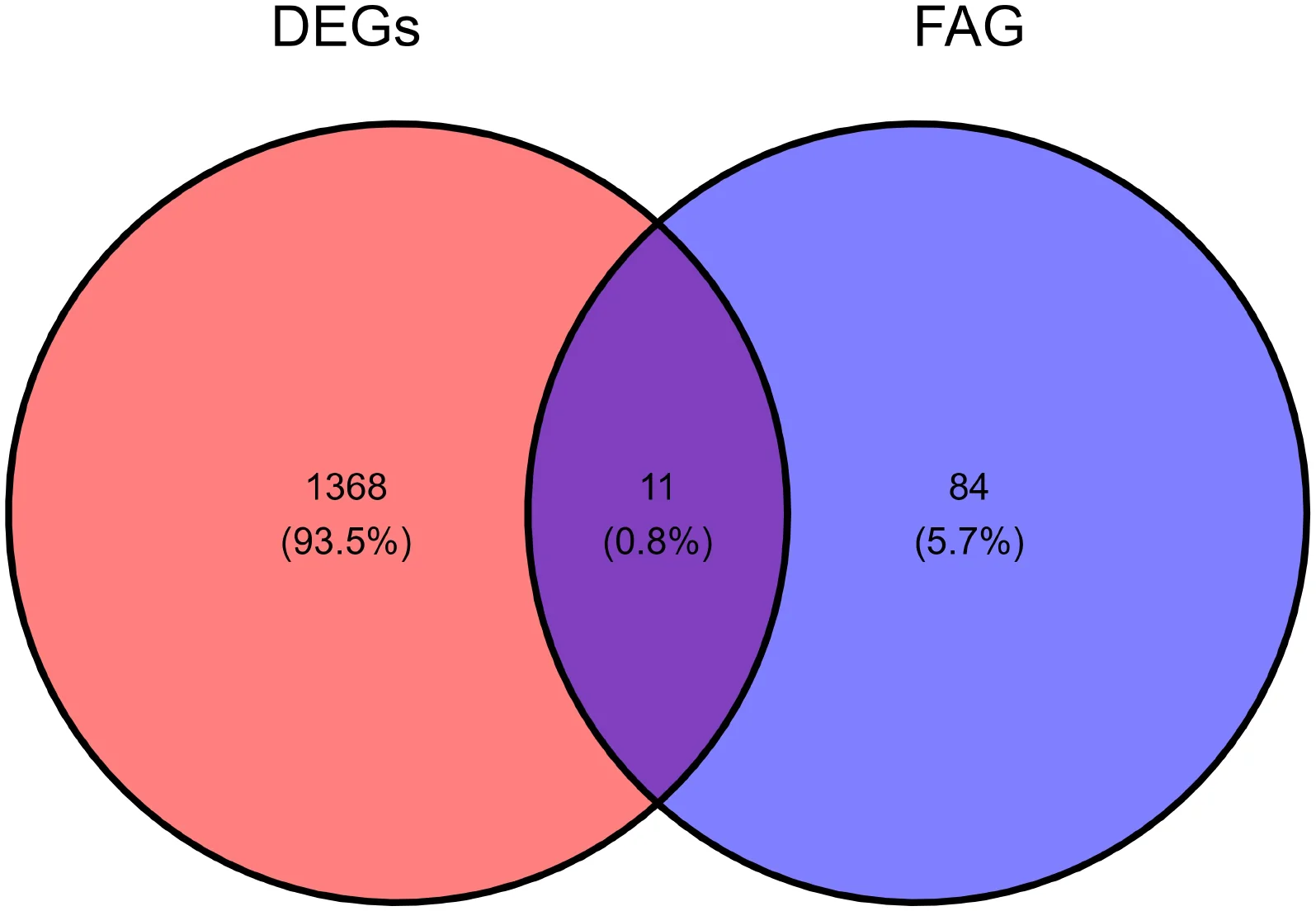
Ferro-aging related differentially expressed genes. DEGs, differentially expressed genes; FAG, ferro-aging-related gene.

GO and KEGG enrichment analysis were used to further explore the relationships among BP, CC, MF, and KEGG pathways of the 11 FAGDEGs in PD (**Figure 4**). The BP analysis results (**Figure 4A**) indicated that FAGDEGs were mainly enriched in biological processes such as cellular response to biotic stimulus, T cell differentiation, and lymphocyte differentiation. The CC results (**Figure 4B**) showed that FAGDEGs were associated with the endoplasmic reticulum lumen, melanosome, and pigment granule. The MF results (**Figure 4C**) demonstrated that FAGDEGs were related to growth factor receptor binding and cytokine activity. The KEGG pathway enrichment analysis (**Figure 4D**) revealed that FAGDEGs were primarily involved in signaling pathways such as the C-type lectin receptor signaling pathway, TNF signaling pathway, NOD-like receptor signaling pathway, Pertussis, and IL-17 signaling pathway.

**Figure 4:**
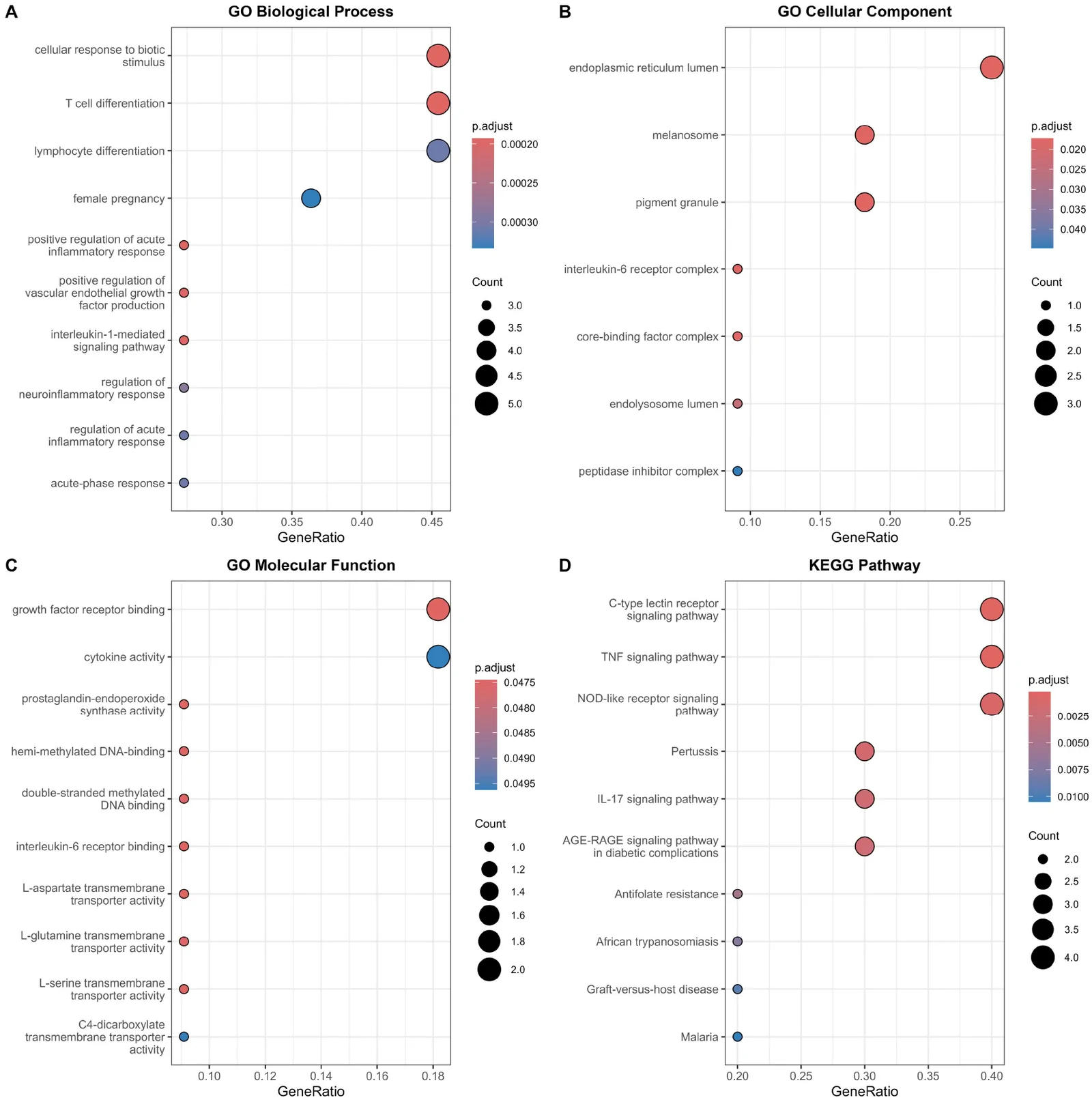
Functional enrichment analysis of ferro-aging related differentially expressed genes. **(A)** GO Biological Process. **(B)** GO Cellular Component. **(C)** GO Molecular Function. **(D)** KEGG Pathway.

### 3.4 WGCNA module detection and analysis results

Using WGCNA, the module most strongly associated with PD was identified. Based on scale independence and average connectivity (**Figure 5A**), a soft threshold β=8 (R²=0.9) was selected. A total of 4,033 genes were included in this analysis. The correlation modules were classified based on the Topological Overlap Matrix (TOM), and modules with a module eigengene (ME) correlation > 0.75 (equivalent to a dissimilarity of < 25%) and containing fewer than 50 genes were merged into larger modules, finally yielding 8 distinct modules. The number of genes assigned to each module was as follows: red (1,067 genes), blue (980), black (485), tan (468), brown (356), grey (303), greenyellow (268), and salmon (106) (detailed gene lists for each module are provided in **Supplementary Table 1**). The grey module represented unassigned genes and was excluded from subsequent trait correlation analyses. Subsequently, correlation analysis between each module eigengene and clinical features was performed (**Figure 5B**). According to the results, the red module (r = 0.67, p < 0.001) and blue module (r = -0.41, p < 0.001) were selected for further analysis. **Figure 5C** shows the gene hierarchical clustering tree and module division. The upper dendrogram is based on TOM matrix clustering, with the vertical axis representing gene dissimilarity; the lower color bands represent the initial modules from dynamic cutting and the final modules after merging (ME correlation > 0.75). Gray indicates unassigned genes.

**Figure 5:**
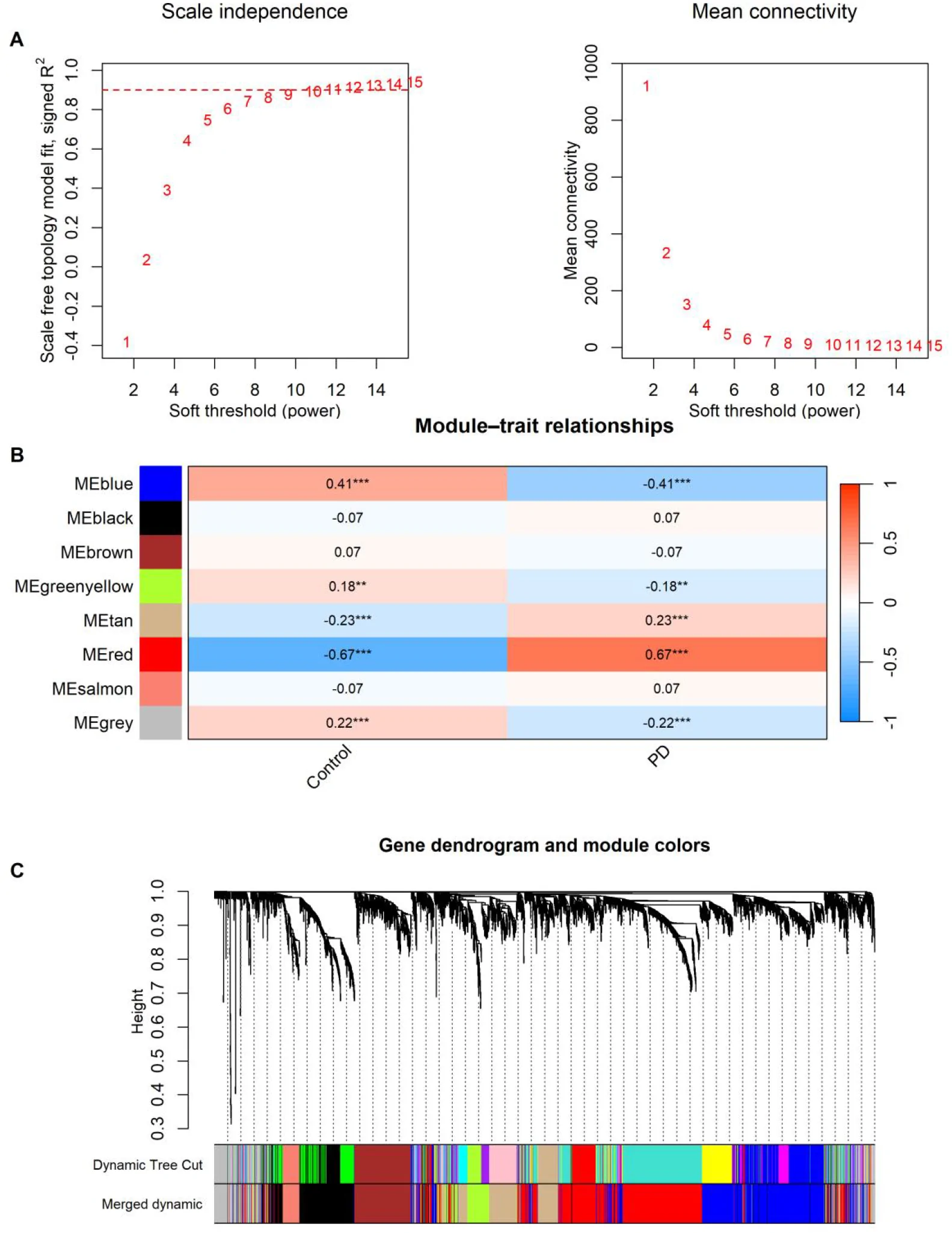
WGCNA analysis. **(A)** Screening of the optimal soft threshold power. **(B)** Heatmap of the correlation between module genes and clinical characteristics. **(C)** Geneclustering dendrogram. PD, periodontitis.

### 3.5 Screening of key genes for ferro-aging

The core genes from the red and blue modules obtained by WGCNA were intersected with DEGs, thereby identifying 1078 module differential genes associated with PD (**Figure 6A**). Based on the intersection of DEGs, core genes from the red and blue modules, and FAG, 7 key genes related to ferro-aging in PD was identified, namely: IRF1, SLC1A5, PTGS2, TXNIP, PDE4B, IL1B, and IL6 (**Figure 6B**).

**Figure 6:**
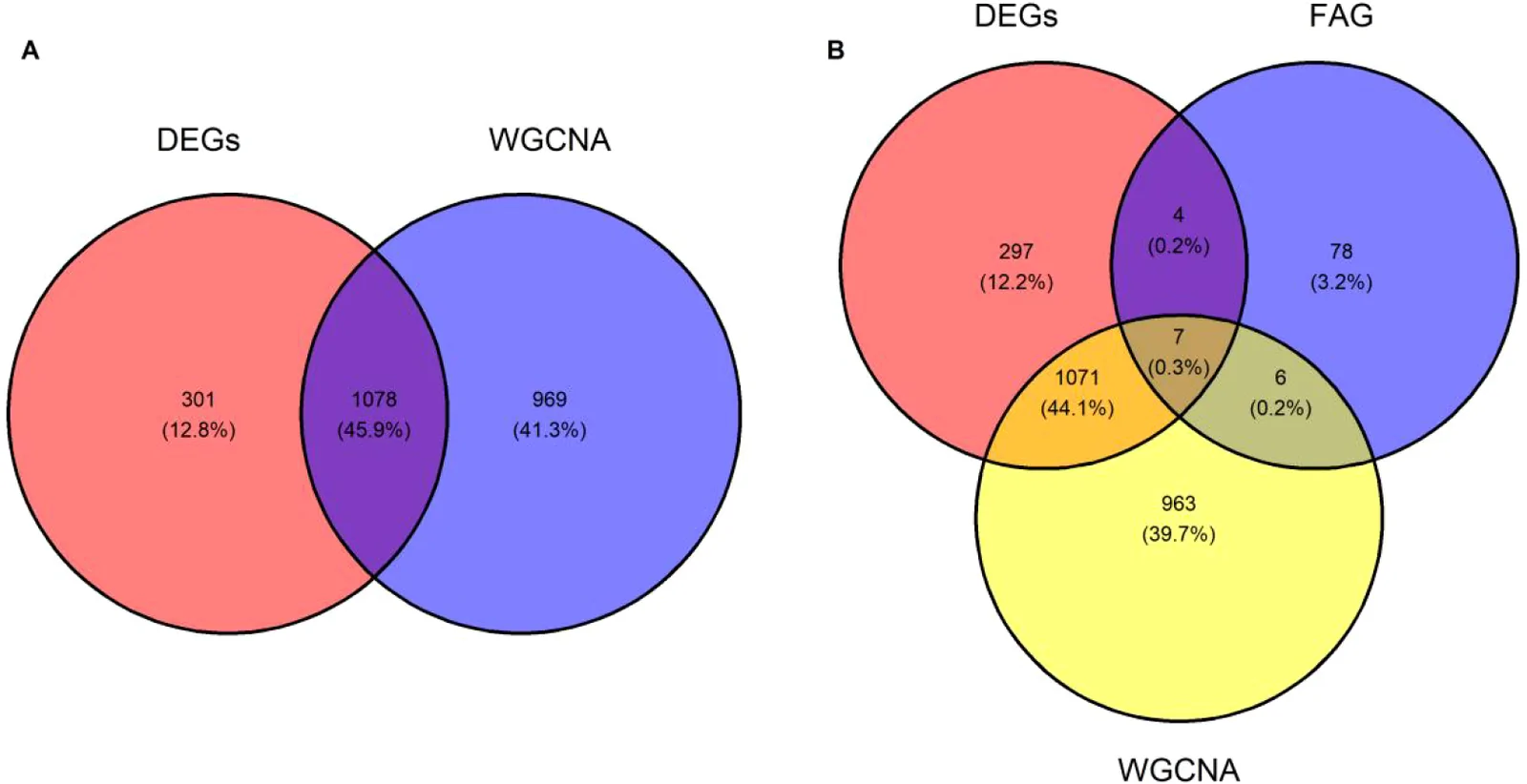
Screening of key genes for ferro-aging. **(A)** The intersection of differentially expressed genes and WGCNA module genes. **(B)** Intersections between differentially expressed genes, WGCNA module genes, and ferro-aging-related genes. DEGs, differentially expressed genes; FAG, ferro-aging-related gene.

### 3.6 Machine learning further identified key core genes of ferro-aging

To prioritize the 7 candidate genes from Section 3.5 for diagnostic utility, we applied LASSO and RF algorithms using their expression profiles across all samples as input and PD status as the binary outcome. LASSO with 10-fold cross-validation selected 4 genes under the lambda.1se criterion (**Figures 7A, B**), while RF ranked gene importance by MeanDecreaseGini and retained all 7 genes (**Figure 7C**). The intersection of both methods yielded 4 overlapping genes (**Figure 7D**): IRF1, SLC1A5, PDE4B, and IL1B, defined as the key ferro-aging-related genes (KFAGs) for further model construction.

**Figure 7:**
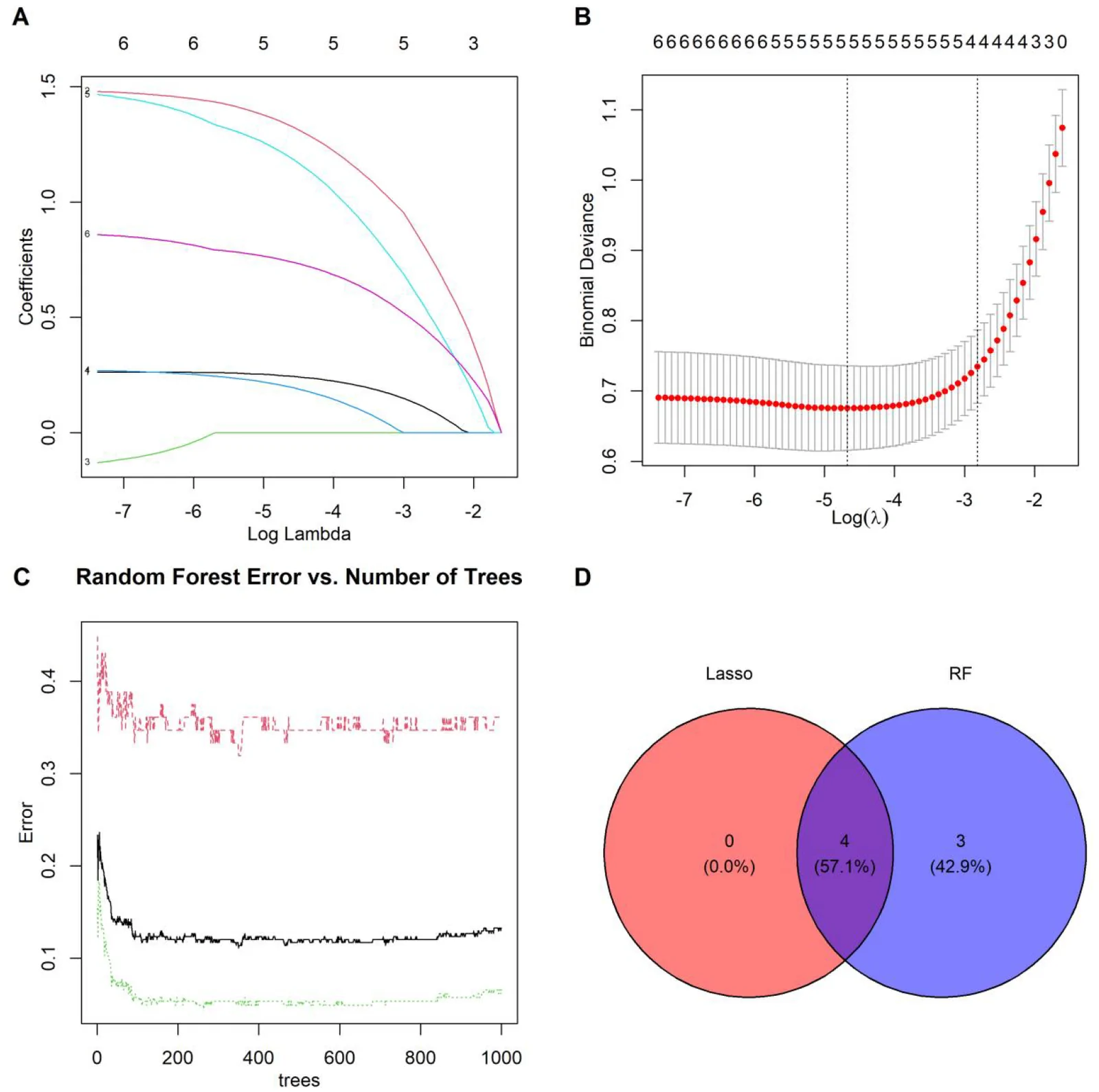
Machine learning further identifies key core genes of ferro-aging. **(A, B)** LASSO regression analysis results. **(C)** RF analysis results. **(D)** Intersection of genes screened by LASSO and RF machine learning.

### 3.7 Development and validation of the PD diagnostic model

Based on 4 KFAGs, we constructed a multi-gene predictive model (**Figure 8A**). This model demonstrated good performance, with an AUC value of 0.897 (**Figure 8B**). The predictive reliability of the model was supported by the calibration curve (**Figure 8C**). Furthermore, the DCA curve (**Figure 8D**) indicated that decisions based on this model may provide additional benefits for PD patients.

**Figure 8:**
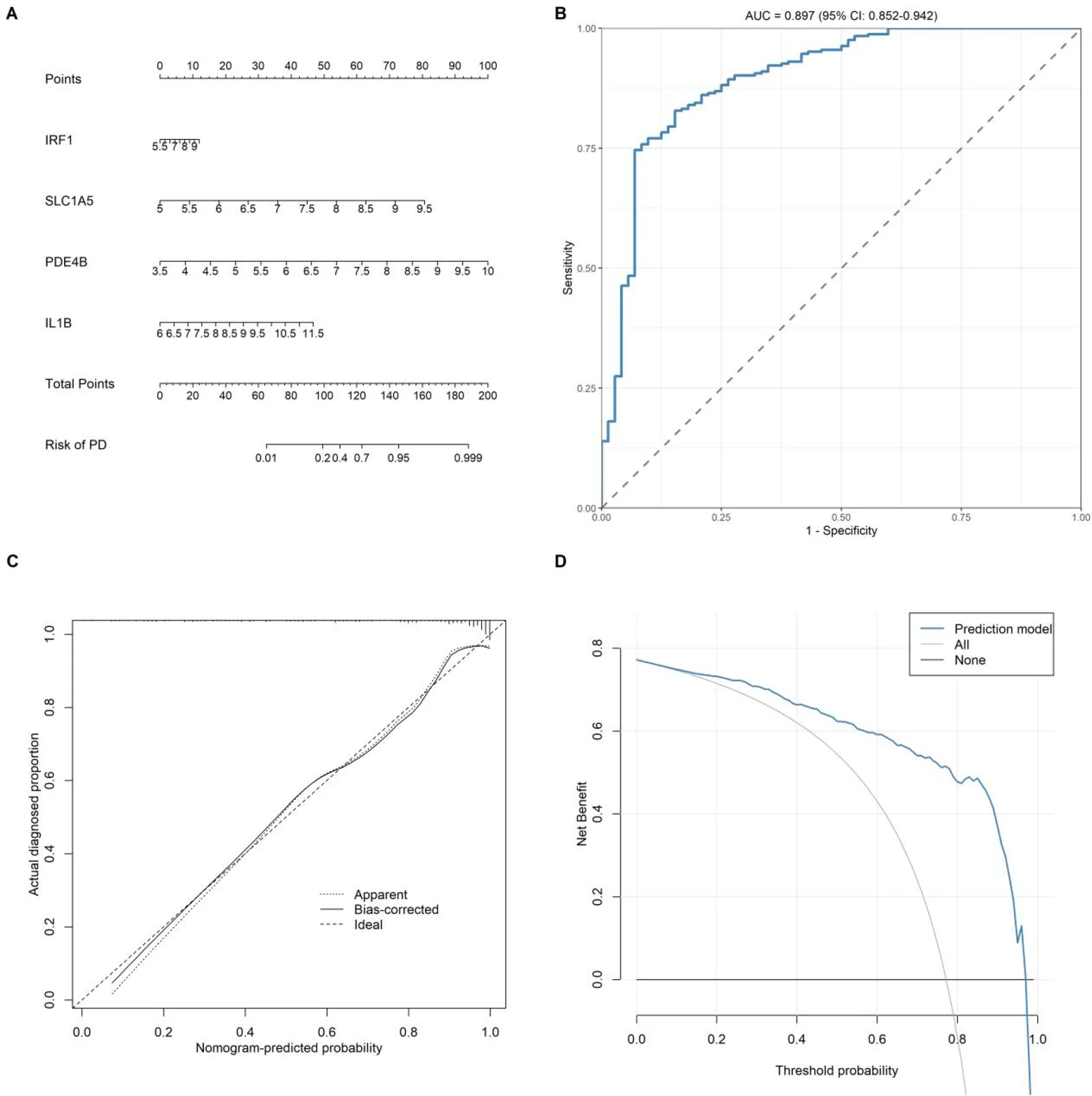
Construction of a nomogram for the diagnostic model of periodontitis. **(A)** Constructing a nomogram based on the key core genes of ferro-aging. **(B)** ROC curve of the nomogram diagnostic model. **(C)** Calibration curve of the nomogram model. **(D)** Decision curve of the nomogram model.

ROC curves and intergroup comparison plots were drawn based on the expression levels of the four ferro-aging-related key core genes (**Figures 9**, **10**). The ROC results showed that the predictive AUC values of all four ferro-aging-related key core genes were greater than 0.6, indicating that they could successfully distinguish PD from healthy samples, demonstrating good diagnostic performance. Moreover, their expression in PD was significantly higher than in healthy tissues (P < 0.05). The expression levels of four KFAGs were validated in the GSE10334 dataset, and the visualization results were also presented using ROC curves and intergroup comparison plots (**Figures 11**, **12**). Therefore, IRF1, SLC1A5, PDE4B, and IL1B were identified as PD-related KFAGs.

**Figure 9:**
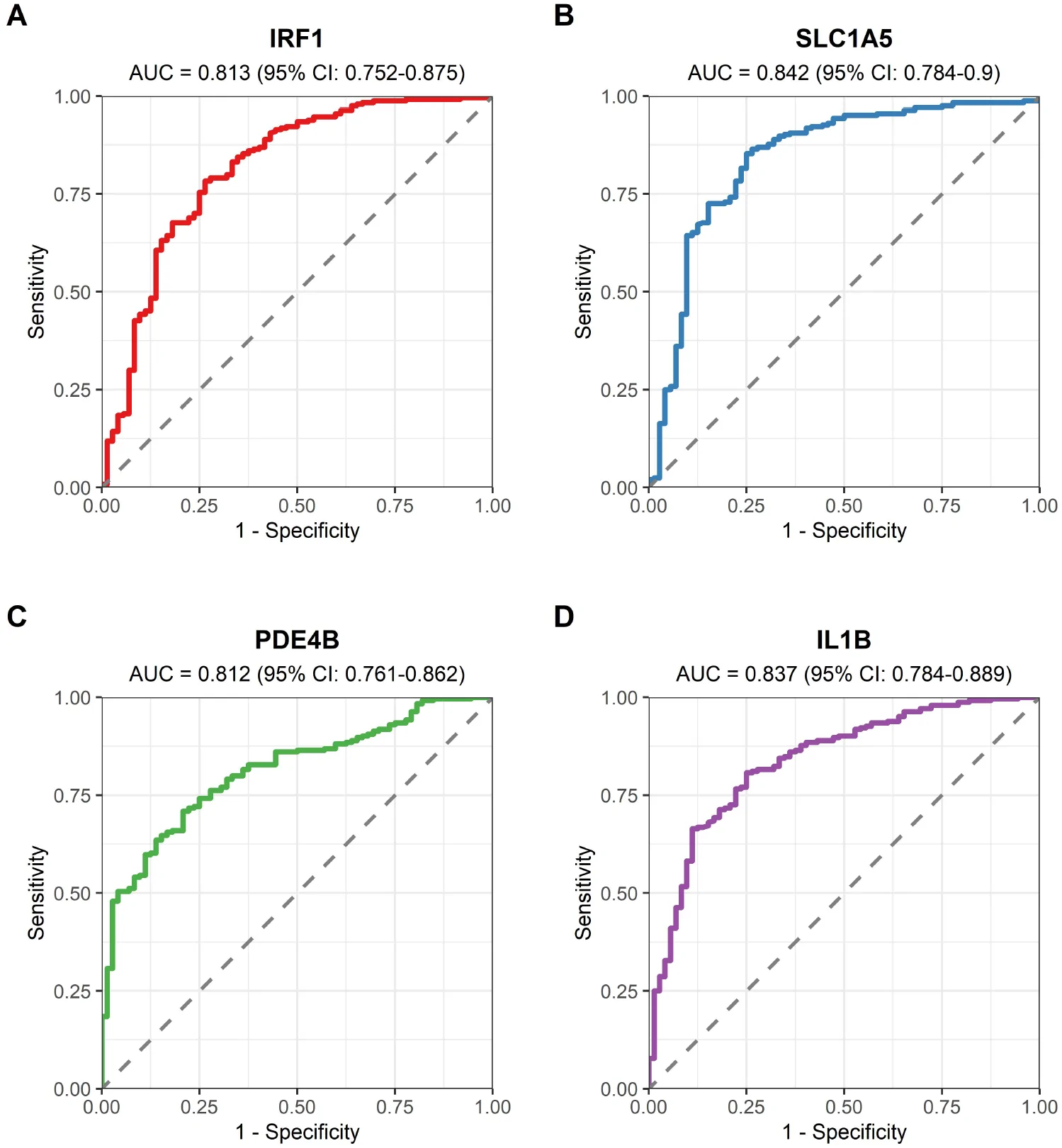
ROC curve analysis of IRF1, SLC1A5, PDE4B, and IL1B in the merged dataset. **(A)** IRF1. **(B)** SLC1A5. **(C)** PDE4B. **(D)** IL1B.

**Figure 10:**
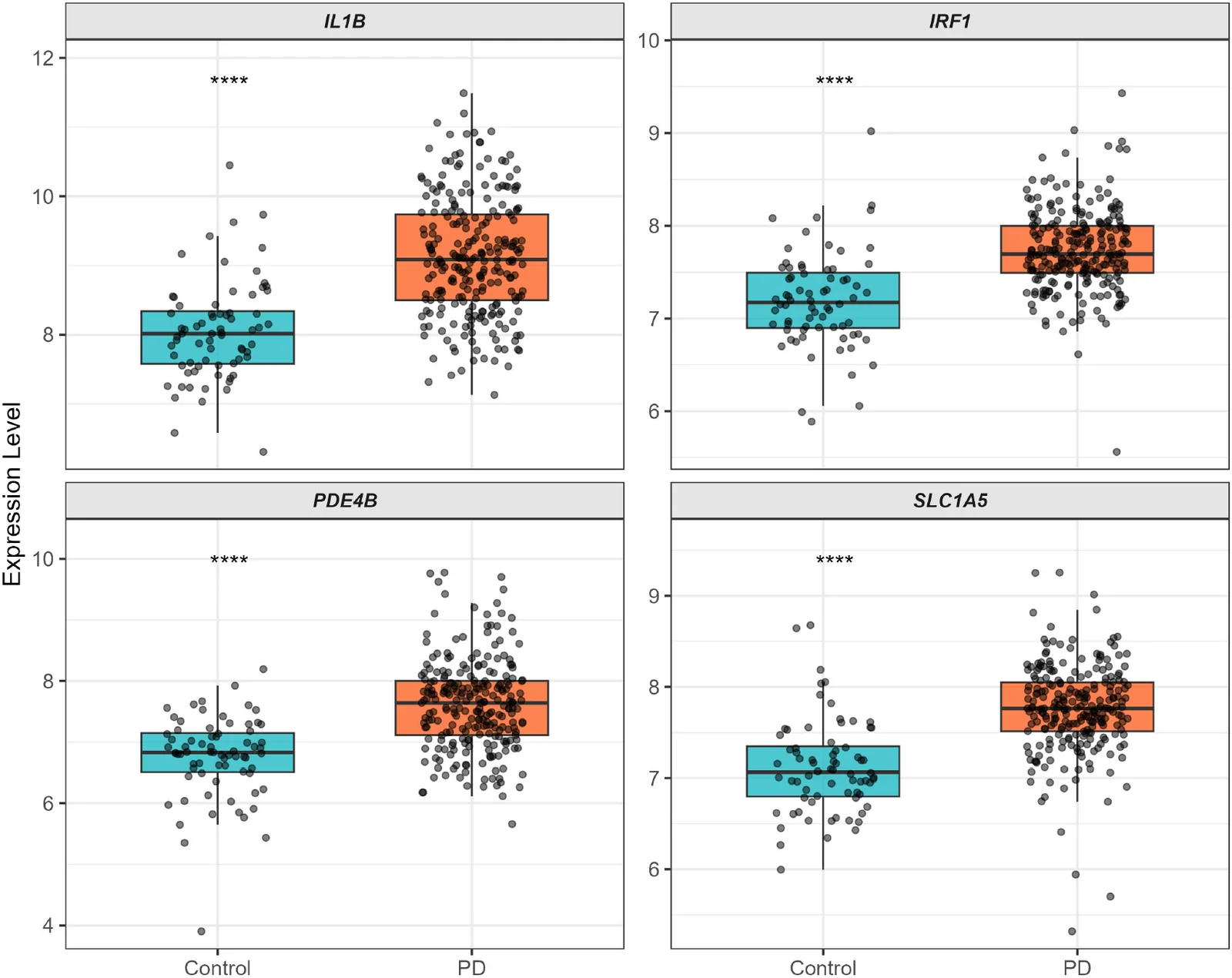
Expression levels of IRF1, SLC1A5, PDE4B, and IL1B in the merged dataset. PD, periodontitis.

**Figure 11:**
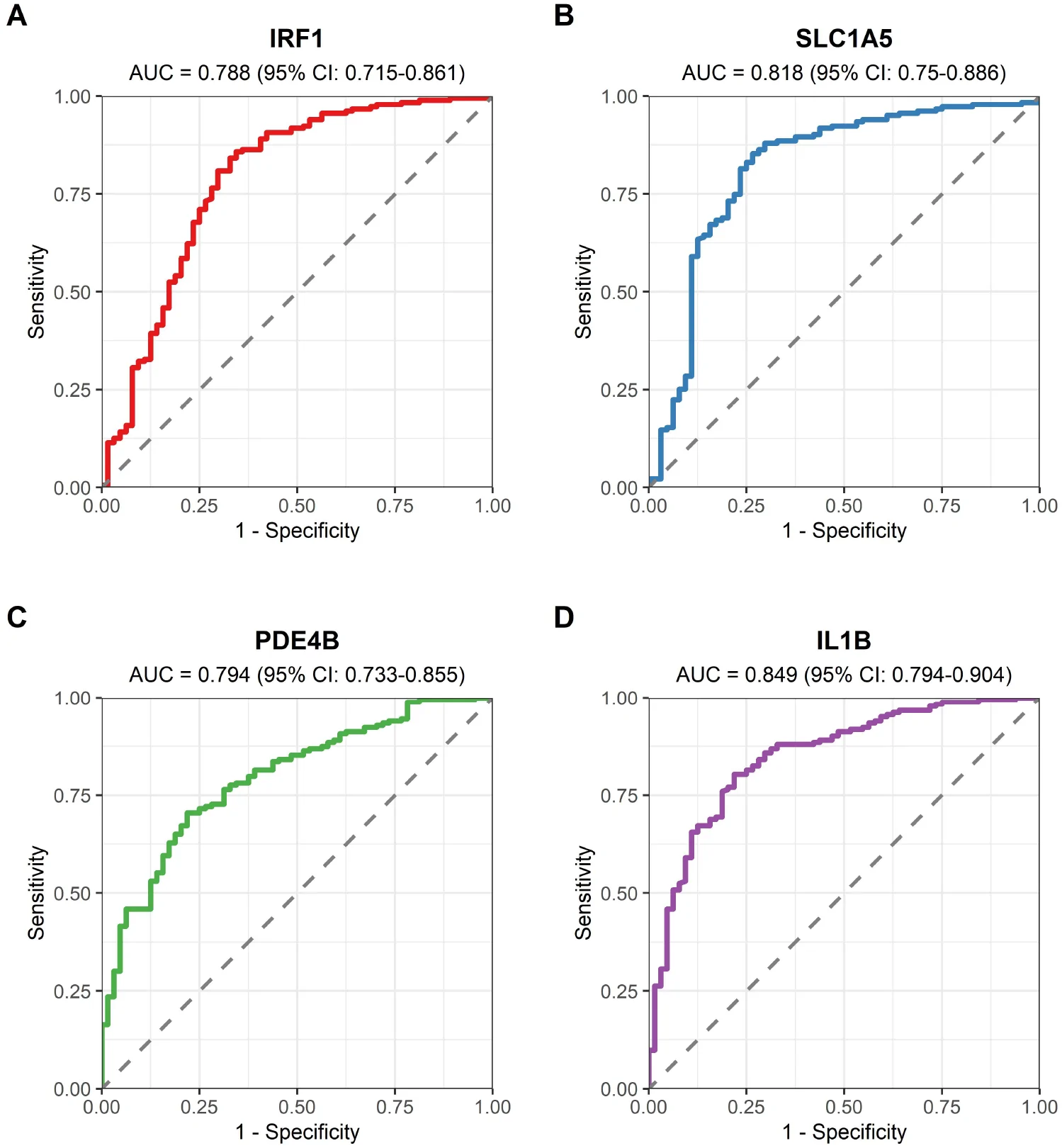
ROC curve analysis of IRF1, SLC1A5, PDE4B, and IL1B in the validation dataset (GSE10334). **(A)** IRF1. **(B)** SLC1A5. **(C)** PDE4B. **(D)** IL1B.

**Figure 12:**
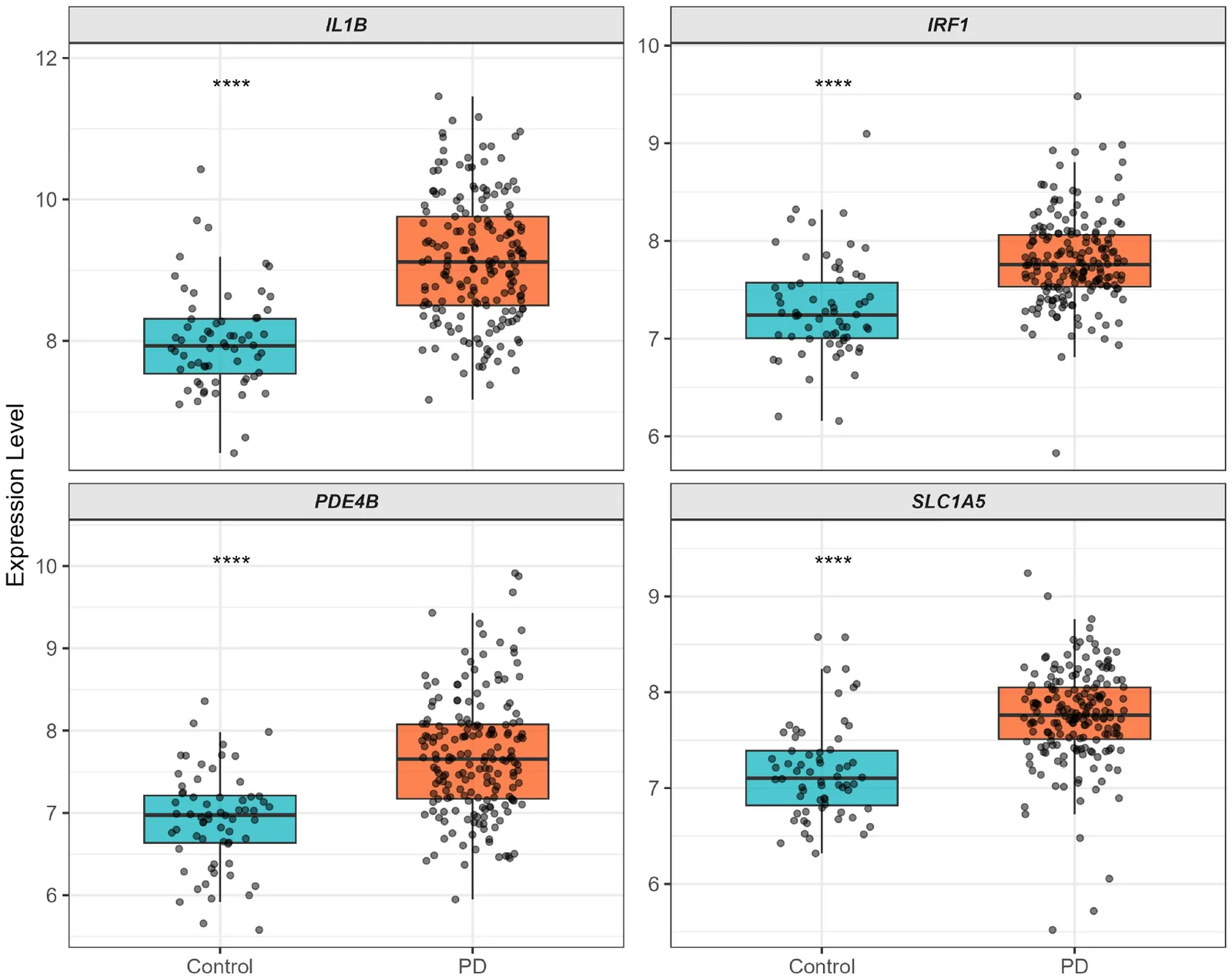
Expression levels of IRF1, SLC1A5, PDE4B, and IL1B in the validation dataset (GSE10334). PD, periodontitis. .

### 3.8 Immune cell infiltration analysis

Based on the merged PD dataset, we quantified the infiltration levels of 22 immune cell subtypes using the ssGSEA algorithm and compared the immune cell infiltration patterns between the PD and control groups, with results visualized as box plots and correlation heatmaps (**Figure 13**). As shown in **Figure 13A**, significant differences in immune cell composition were found between PD samples and control samples. In PD, the expression levels of Type 1 T helper (Th1) cells, Activated B cells, Activated CD4 T cells, Activated dendritic cells, Natural killer cells, Type 17 T helper cells, and other immune cells were significantly elevated. Among them, IRF1 and SLC1A5 are negatively correlated with the response of certain immune cells, such as Th2 cells. Moreover, strong interrelationships were observed among various immune cell types (**Figure 13B**). Based on the correlation analysis between the four PD-related KFAGs and immune cells, the results showed a strong association between KFAGs and immune cells (**Figure 13C**).

**Figure 13:**
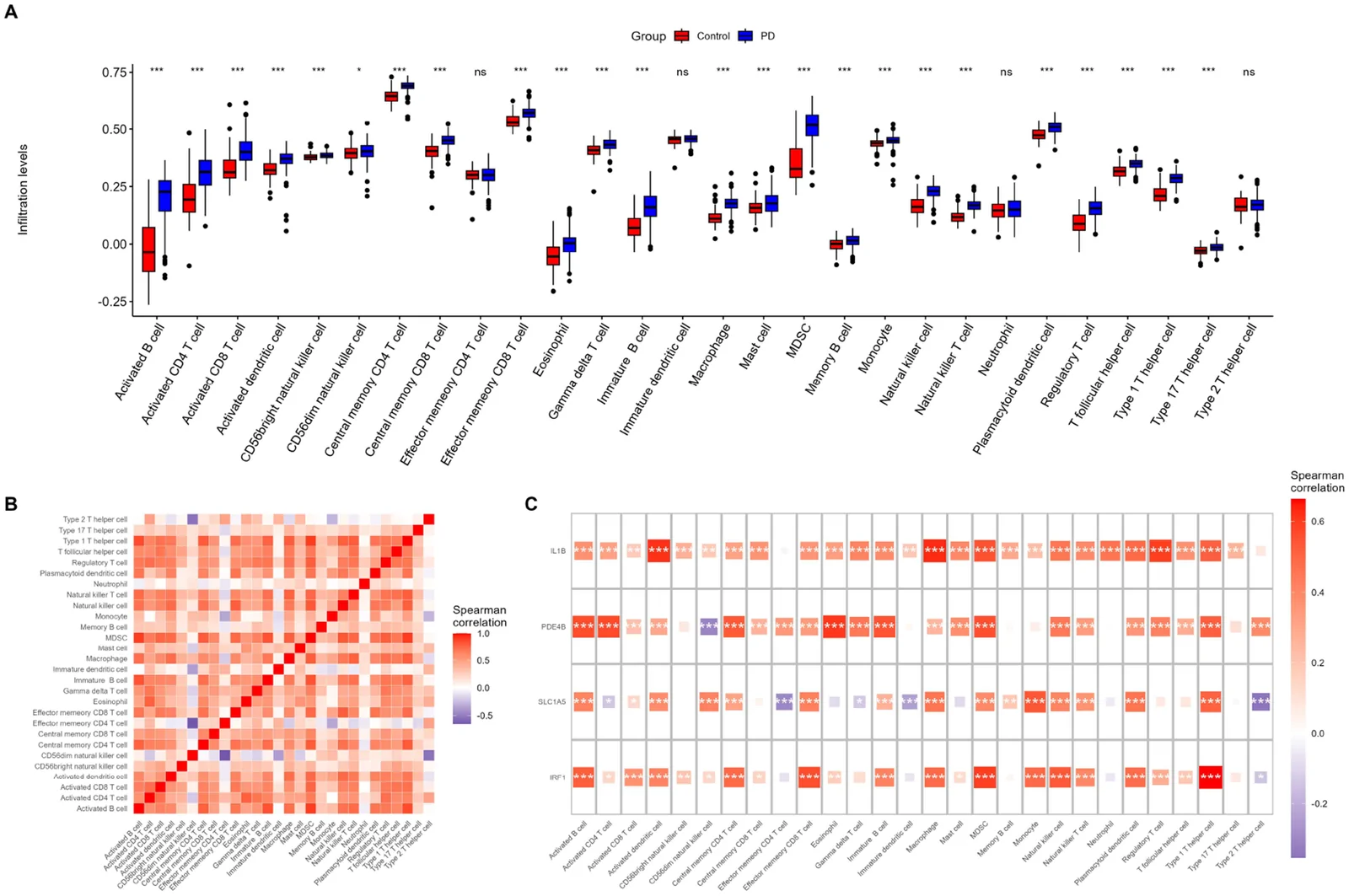
Immune infiltration analysis results. **(A)** Immune cell expression between control and PD groups. **(B)** Correlations among immune cells. **(C)** Correlation between immune cells and KFAGs. PD, periodontitis. KFAGs, key ferro-aging-related genes, ns, not significant; *, p < 0.05; **, p < 0.01; ***, p < 0.001.

### 3.9 RT-qPCR validation of key core genes

To validate the bioinformatic analysis results, we performed RT-qPCR on 6 clinical gingival tissue samples (3 PD patients and 3 healthy controls). As shown in **Figure 14**, compared with the control group, all four KFAGs in the PD group showed statistically significant differences. The expression levels of IRF1, SLC1A5, PDE4B, and IL1B in the PD group were significantly higher than those in the control group, and their expression trends were consistent with the bioinformatic analysis results.

**Figure 14:**
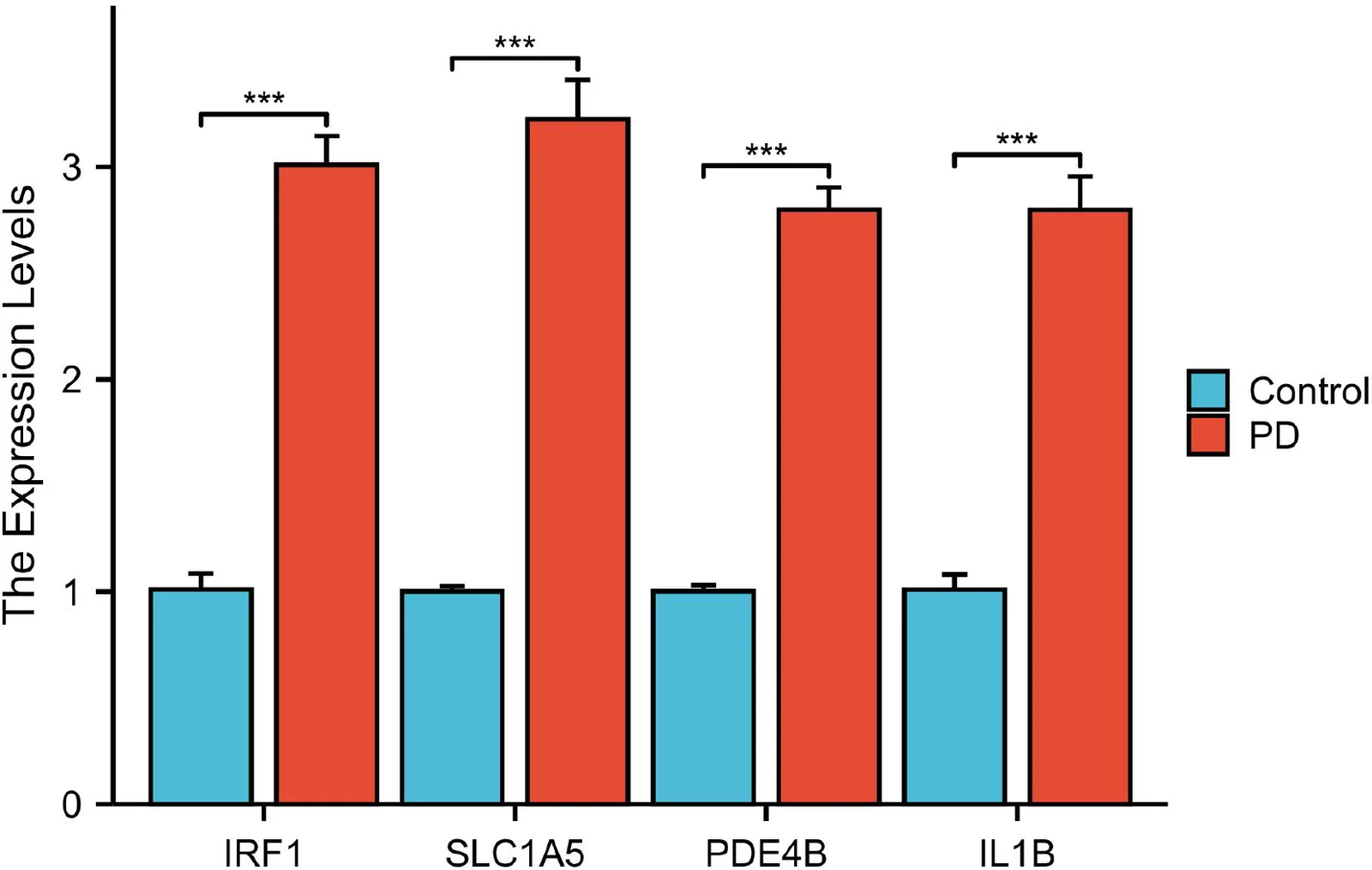
Analyze the expression levels of IRF1, SLC1A5, PDE4B, and IL1B through qRT-PCR. PD, periodontitis, *** indicates p < 0.001.

## 4 Discussion

PD is a typical chronic inflammatory disease initiated by dental plaque biofilm, leading to the destruction of periodontal supporting tissues. Its pathological features include elevated ROS, mitochondrial dysfunction, lipid peroxidation, and release of pro-inflammatory factors (Papadakis and Doufexi, 2025; Huang et al., 2026). In PD, red blood cells released from gingival bleeding break down and release large amounts of free iron (Mukherjee, 1985; Subbaiah et al., 2022). These iron ions catalyze the production of highly reactive free radicals, triggering intense oxidative stress that directly damages periodontal tissues. Based on this microenvironment, periodontal ligament fibroblasts, osteoblasts, and other cells may undergo ferroptosis or enter a state of aging, subsequently secreting large amounts of SASP factors, continuously recruiting inflammatory cells, degrading collagen, and inhibiting osteogenesis, resulting in irreversible bone loss (Masi et al., 2011; Xia et al., 2017).

Ferroptosis, as an acute cell death event, is more suitable for explaining focal and rapid tissue necrosis, but it is difficult to fully account for the slow, diffuse, and decades-long tissue destruction characteristic of PD. This limitation in theoretical applicability suggests that ferroptosis may not be the core mechanism driving the chronic progression of PD. Therefore, it is urgent to construct a new theoretical framework to elucidate the pathological progression mechanism of PD. In recent years, “ferro-aging” has been proposed as an iron-dependent chronic aging mechanism, characterized by long-term iron accumulation and lipid peroxidation-induced progressive decline in cellular function, impaired tissue regeneration, and systemic aging. Notably, the typical pathological manifestations of PD—decades-long, slowly worsening alveolar ridge loss and soft tissue destruction—precisely align with the chronic, progressive temporal features of ferro-aging. Thus, PD may serve as an ideal model for studying the mechanisms of ferro-aging and its clinical interventions.

In this study, we integrated two independent datasets (GSE16134 and GSE23586) from the GEO database and initially identified 11 FAGDEGs. GO and KEGG enrichment analyses showed that these genes are mainly involved in responses to biostimuli, T cell differentiation, growth factor receptor binding, and cytokine activity, and are associated with the C-type lectin receptor signaling pathway, TNF signaling pathway, NOD-like receptor signaling pathway, and IL-17 signaling pathway. Subsequently, using WGCNA and machine learning algorithms, four KFAGs were identified: IRF1, SLC1A5, PDE4B, and IL1B. ROC analysis suggested that these four KFAGs have high diagnostic value for PD, and their expression is significantly upregulated in PD. The GSE10334 dataset also validated these findings. Furthermore, immune infiltration analysis revealed a strong positive correlation between these four KFAGs and immune cells such as dendritic cells, monocytes/macrophages, neutrophils, T lymphocytes, and B lymphocytes, which play crucial roles in the pathological immune response of PD. Specifically, IRF1 and SLC1A5 were positively correlated with Th1 cell responses but negatively correlated with Th2 cell responses. It has been reported that pathogens such as *Porphyromonas gingivalis* (*P. gingivalis*) activate Th1 cell, causing them to secrete large amounts of pro-inflammatory factors such as IFN-γ and TNF-α. These factors, on the one hand, activate macrophages and promote osteoclast differentiation, directly leading to alveolar bone resorption; on the other hand, they inhibit Th2 cell function through the IFN-γ-mediated signaling pathway, creating a Th1/Th2 imbalanced immune microenvironment. Therefore, PD exhibits typical Th1-dominant immune response characteristics locally. These combined results indicate that these KFAGs play an important role in the immune process of PD.

Interferon Regulatory Factor 1 (IRF1) is a crucial member of the interferon regulatory factor family, functioning as a transcription factor that plays a key role in regulating immune responses and inflammatory reactions. IRF1 is primarily activated through the JAK/STAT signaling pathway and participates in immune responses mediated by type I (IFN-α/β) and type II (IFN-γ) interferons (Ravi Sundar Jose Geetha and Fischer, 2024; Zhu et al., 2026). This study observed that in the lesioned tissues of patients with PD, the expression level of IRF1 was significantly elevated and positively correlated with the activation of Th1 cell. This finding is consistent with previous reports indicating that the PD context can promote the upregulation of IRF1 expression (Liu et al., 2022). However, some *in vitro* studies have yielded seemingly contradictory conclusions (Rodriguez-Hernandez and Sokoloski, 2021). Scholars such as De Andrade K Q and Jauregui C E reported that *P. gingivalis* can suppress IRF1 expression in gingival epithelial cells or fibroblasts (Jauregui et al., 2013; de Andrade and Almeida-da-Silva, 2019). As barrier cells of periodontal tissues, gingival epithelial cells and fibroblasts, when exposed to live bacteria, may experience direct degradation of STAT1/IRF1 proteins or inhibition of their transcription through bacterial secretion of proteases, thereby facilitating local immune evasion (Fitzpatrick et al., 2009; Hajishengallis, 2011; Jauregui et al., 2013). Notably, Elkaim R et al. found in a cell model that while live bacteria significantly reduced IRF1 mRNA and protein levels, treatment with purified *P. gingivalis* lipopolysaccharide (LPS-Pg) significantly increased IRF1 expression (Elkaim et al., 2017). This “live bacteria-LPS” discrepancy suggests that the regulation of IRF1 expression may depend on the form of bacterial presence and its interaction mode with host cells. Studies on other inflammatory diseases have revealed that when bacterial components, especially LPS, dominate, they can induce antigen-presenting cells to produce IL-12, thereby promoting the differentiation of naïve CD4^+^^+^ T cells into Th1 cells (Salkowski et al., 2000; Ohmori and Hamilton, 2001). Simultaneously, IL-12 and IFN-γ synergistically activate the STAT1 signaling pathway, upregulating IRF1 expression (Yan et al., 2021). As a transcription factor, IRF1 can further promote the production of Th1-type chemokines and IFN-γ, thereby amplifying Th1-type inflammatory responses. This mechanism, triggered by LPS and mediated through the IL-12-IFN-γ-STAT1 axis, ultimately driving Th1 immune responses, has been confirmed in various inflammatory diseases. Although this mechanism still requires direct validation in periodontitis, combined with the finding in this study that IRF1 expression is positively correlated with Th1 responses, we speculate that in the complex lesioned tissues of PD, pathogen-associated molecular patterns derived from the microbiota (such as LPS) may drive infiltrating immune cells to highly express IRF1 through a similar signaling axis, thereby promoting the amplification of local Th1-type inflammatory responses.

SLC1A5 (solute carrier family 1 member 5), commonly known as ASCT2, encodes a sodium-dependent neutral amino acid transporter and serves as the primary channel for glutamine uptake in mammalian cells (Kawakami et al., 2022). In the regulation of cellular redox homeostasis, SLC1A5-mediated glutamine uptake constitutes the rate-limiting step for glutathione (GSH) biosynthesis (Wu et al., 2026). Given that GSH is an essential cofactor for glutathione peroxidase 4 (GPX4) in clearing lipid peroxides, the functional status of SLC1A5 may indirectly determine cellular sensitivity to iron-dependent oxidative damage, thereby participating in the occurrence of ferroptosis or ferro-aging (Raj Rai et al., 2021). Consistent with previous research findings (Zhang et al., 2022; Chen et al., 2024), this study further confirmed a significant upregulation of SLC1A5 transcription levels in inflamed periodontal tissues. This aberrant expression pattern may play multiple roles in the pathogenesis of PD, encompassing key aspects such as ferroptosis regulation, immunometabolic reprogramming, and osteoimmunological coupling (Zhang et al., 2022; Ebersole et al., 2024; Asa'ad and Hasuike, 2026). Notably, this study found that SLC1A5 was significantly upregulated in PD tissues, with its expression levels closely correlated with the degree of immune cell infiltration, particularly involving myeloid and lymphoid cells (e.g., macrophages, neutrophils, and lymphocytes). Given the necessity of glutamine supply for the activation, proliferation, and effector functions of T cells and macrophages, aberrant SLC1A5 expression may participate in the course of PD through immunometabolic reprogramming.

PDE4, as a subfamily of phosphodiesterases (PDEs) that specifically hydrolyzes cAMP, includes four isozymes: A, B, C, and D (Sherstnev et al., 2025). It is widely distributed in immune cells and inflammatory cells, and its expression level is closely related to the activation state of the inflammatory response. Among the PDE4 subtypes, PDE4B has attracted significant attention due to its predominant expression in immune cells (such as macrophages, neutrophils, and lymphocytes) and its specific regulation of inflammatory signals (Dhar et al., 2025). Existing clinical studies often target “PDE4” as a whole, showing significantly upregulated expression in PD (Liu et al., 2022), but there is no independent quantitative data on the expression level of the PDE4B subtype in PD patients. This study found that PDE4B expression is significantly upregulated in PD and is associated with various immune cells. Considering the predominant expression characteristics of PDE4B in immune cells and the fact that PD is essentially an inflammatory disease characterized by immune cell infiltration and activation, it is reasonable to infer that PDE4B may be the main contributing subtype to the upregulated expression of PDE4 in periodontal tissues.

Periodontal pathogen infection can induce the formation of a microenvironment characterized by both iron overload and inflammation in local tissues, thereby driving the transcriptional upregulation of the IL1B gene (Wang et al., 2023; Bao et al., 2024). Interleukin-1β (IL-1β), encoded by the IL1B gene, is the most important secreted pro-inflammatory cytokine in the IL-1 family, and its sustained high expression is one of the core driving factors for periodontal tissue destruction in PD (de Alencar et al., 2020). In the pathological context of PD, the functional link between the ACSL4 inhibitory activity of rosiglitazone and its regulatory effect on IL-1β warrants in-depth investigation. In an orthodontic-induced inflammatory root resorption model, periodontal ligament cells subjected to combined compressive stress and IL-1β stimulation showed significantly elevated ACSL4 expression levels and Fe²^+^ concentrations (Liu et al., 2025); these phenotypes were effectively reversed by intervention with the ACSL4 inhibitor rosiglitazone. This study suggests the existence of a causal chain of “IL-1β stimulation—ACSL4 upregulation—iron overload pathway activation” at the periodontal tissue level, indicating that rosiglitazone, as an ACSL4 inhibitor, can inhibit ACSL4 expression and iron overload at the downstream level of the pathway, thereby blocking the process of ferro-aging. Meanwhile, multiple animal experiments have consistently shown that rosiglitazone significantly reduces the protein expression level of IL-1β in periodontal tissues in experimental PD, promotes M2 macrophage polarization, increases the expression of the anti-inflammatory factor IL-10, and decreases the M1/M2 macrophage ratio (Qiao et al., 2018; Lina, 2025; Zhou et al., 2026). Therefore, it is inferred that IL-1β and ACSL4 may constitute an upstream-downstream regulatory axis in the ferro-aging process of PD, and rosiglitazone, due to its unique dual-target pharmacological properties, can intervene in this pathological pathway at both the upstream (inhibiting IL-1β production) and downstream (inhibiting ACSL4 activity) nodes, thereby demonstrating a more comprehensive periodontal protective potential compared to single-target interventions.

This study has several limitations that warrant acknowledgment. First, as the identification of core genes and immune infiltration analyses relied primarily on transcriptomic data from public repositories, the functional roles of IRF1, SLC1A5, PDE4B, and IL1B in periodontal ferro-aging, along with the causal links between immune cell infiltration and ferro-aging, necessitate further *in vitro* and *in vivo* validation. Second, although we included an independent external validation dataset (GSE10334), this validation remains at the transcriptomic level with retrospective data. Furthermore, RT-qPCR validation was conducted on a small cohort of six clinical tissue samples (3 PD and 3 healthy control samples); consequently, this limited sample size may compromise statistical power and generalizability. Future investigations involving larger clinical cohorts, complemented by animal models and functional assays, are warranted to comprehensively elucidate the molecular mechanisms and translational potential of these biomarkers in periodontal disease.

## 5 Conclusion

In this study, we identified IRF1, SLC1A5, PDE4B, and IL1B as key biomarkers associated with ferro-aging in PD.

## Data Availability

The datasets presented in this study can be found in online repositories. The names of the repository/repositories and accession number(s) can be found below: https://www.ncbi.nlm.nih.gov/geo/, GSE16134, GSE23586, GSE10334.
